# c-FLIP is a target of the E3 ligase deltex1 in gastric cancer

**DOI:** 10.1038/s41419-017-0165-6

**Published:** 2018-01-26

**Authors:** Tzu-Sheng Hsu, Shu-Ting Mo, Ping-Ning Hsu, Ming-Zong Lai

**Affiliations:** 1 Institute of Molecular Biology, Academia Sinica, Taipei, Taiwan; 2 Institute of Immunology, College of Medicine, National Taiwan University, Taipei, Taiwan

## Abstract

The ubiquitin E3 ligase *DELTEX1* (*DTX1*) is specifically downregulated in gastric cancer tissues, and expression of *DTX1* is linked to better prognoses and survival in gastric cancer. Cellular FLICE inhibitory protein (c-FLIP) is known for its pivotal role in the resistance of cancer cells to death receptor-induced cell death. Here, we show that DTX1 is an E3 ligase for c-FLIP in gastric cancer cells. DTX1 promoted c-FLIP downregulation. Overexpression of DTX1 sensitized gastric cancer cells to TRAIL-induced apoptosis, whereas DTX1-knockdown attenuated apoptosis induction. DTX1 binds c-FLIP_L_ and directs it into the endosome-lysosomal pathway for proteasome-independent degradation. Moreover, induction of DTX1 in AGS cells by geldanamycin conferred susceptibility of those cells to TRAIL-induced apoptosis. Our results reveal a tumor-suppressive role for DTX1 and suggest a new approach to increasing TRAIL efficacy by raising DTX1 levels in gastric cancer therapy. DTX1 also enhanced c-FLIP degradation and FasL-induced and TRAIL-induced apoptosis in T cells, suggesting that DTX1 constitutes one of the physiological mechanisms regulating c-FLIP stability.

## Introduction

Gastric cancer cells are characterized by their resistance to apoptosis induction by death receptors. Gastric cancer has one of the world’s leading cancer mortality rates, with a poor 5-year survival rate^1–3^. Advanced stages of gastric cancer show local invasion, peritoneal dissemination, and hepatic or para-aortic lymph node metastasis. Surgery remains the curative therapy, but is limited to non-metastatic gastric cancer. The efficacy of chemotherapy for gastric cancer is poor due to multidrug resistance (MDR). Therefore, identification of novel biomarkers and development of new therapeutics for gastric cancer are one of the demanding priorities.

The Death receptor (DR) agonist TRAIL has been explored for its efficacy to induce apoptosis in different types of cancers^4–6^, including gastric cancer^7,8^. Like other death receptors, engagement of TRAIL receptors (DR4 and DR5) by TRAIL results in the formation of death-inducing signaling complexes (DISC) containing FADD and procaspase-8^9–12^. Procaspase-8 undergoes autoproteolytic cleavage to generate active caspase-8 at DISC, leading to activation of downstream caspases and irreversible cell damage. Cellular FLICE-inhibitory protein (c-FLIP) is a master anti-apoptotic factor that suppresses death receptor-induced apoptosis by interfering with the processing of procaspase-8 at DISC^9–15^. c-FLIP also inhibits necrosis and autophagy^16–18^. c-FLIP is partly accountable for the failure of TRAIL receptor agonists in clinical attempts to treat cancers^4,19^, so it is a target for cancer therapy^19–21^. Expression of c-FLIP is induced by activation signaling, including NF-κB^22^, Akt, and ERK^13,19,22–24^. Levels of c-FLIP protein are subjected to regulation by two ubiquitin E3 ligases, ITCH and CBL, through the promotion of polyubiquitination and subsequent proteosomal degradation of c-FLIP^25,26^.

TRAIL receptors and the downstream effector caspase-8 are intact in gastric cancer cells^27,28^. However, gastric cancers are generally resistant to TRAIL-induced cell death, and induction of TRAIL-mediated cytotoxicity always requires co-stimulation with a sensitizing reagent. c-FLIP is upregulated in gastric cancer and is associated with metastasis and tumor progression^29,30^. As in other types of cancer, c-FLIP contributes to the resistance to TRAIL-induced apoptosis in gastric cancer^31–34^. We have previously shown that *Helicobacter pylori* enhances the susceptibility to TRAIL-induced apoptosis in gastric cancer cells by downregulation of c-FLIP^34^.

Deltex (DTX) is a target of Notch, and is composed of Notch-binding WWE domains at the N-terminus, followed by a proline-rich motif, and a C-terminal RING finger domain^35,36^. DTX1 confers ligand-independent activation of Notch by directing the ubiquitination and endosomal entry of Notch^37,38^. Similar to the E3 ligases Itch and Cbl-b^39^, DTX1 is a target of NFAT and is involved in T cell tolerance^40,41^. We recently found that DTX1 promotes the degradation of PKCθ and PLC-γ in a way similar to ITCH and Cbl-b^42^.

In the present study, we show that DTX1 is specifically downregulated in gastric cancer and is critical for the resistance of gastric cancer cells to TRAIL-induced cell death. DTX1 binds to c-FLIP and promotes degradation of c-FLIP through the endosome-lysosomal pathway. Re-introduction of DTX1 into gastric cancer cells increased TRAIL-induced apoptosis and also reduced c-FLIP. In addition, a treatment that increased DTX1 expression also sensitized gastric cancer to TRAIL treatment. Our results suggest that induction of DTX1 could be a new approach to enhancing the benefits of TRAIL-mediated cancer therapy. We also found that DTX1 enhanced c-FLIP degradation and Fas-induced and TRAIL-induced apoptosis in T cells, indicating that DTX1 constitutes one of the physiological mechanisms regulating c-FLIP stability.

## Results

### DTX1 expression is negatively correlated with gastric cancer progression

**Fig. 1:**
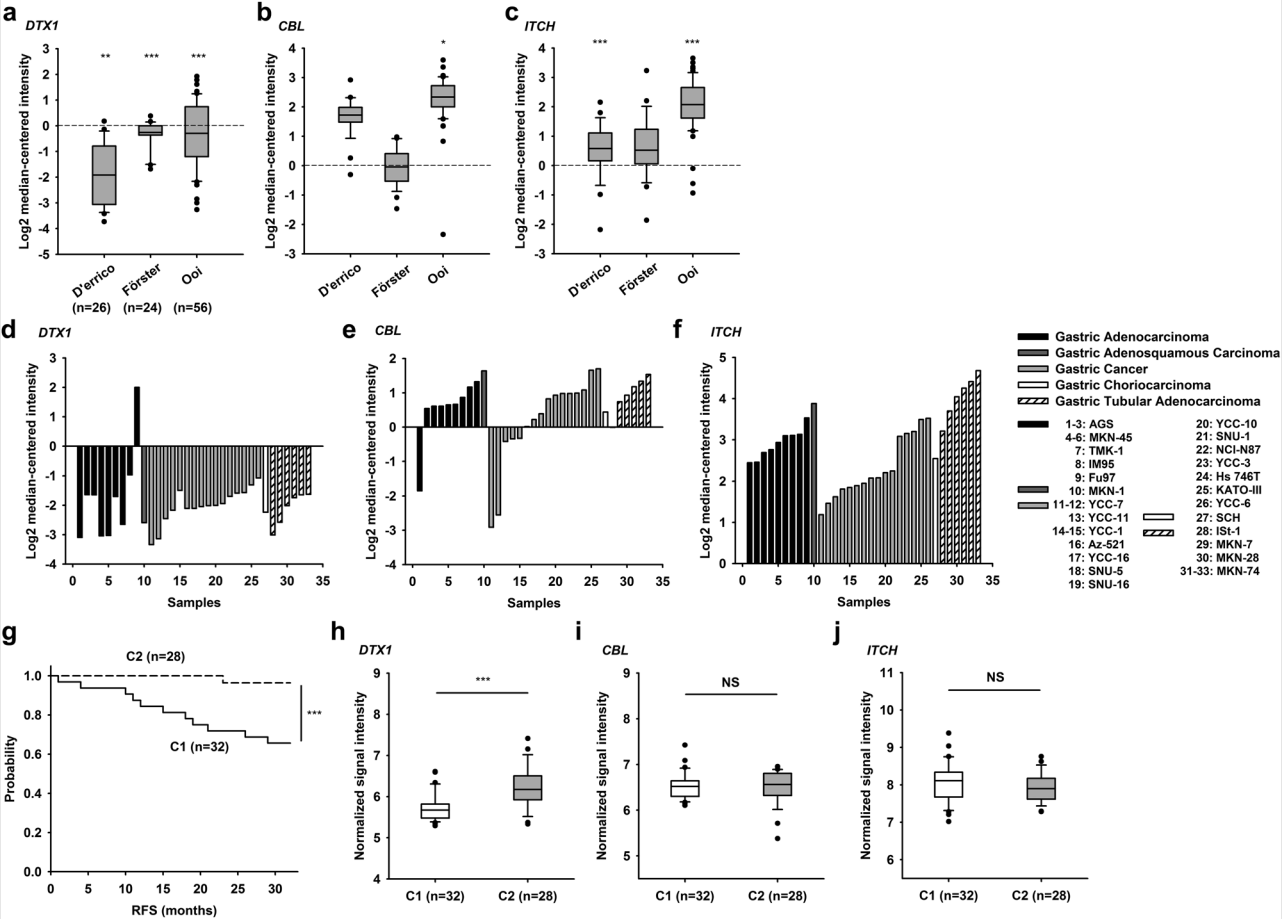
***DTX1*is downregulated in gastric cancer and*DTX1*is correlated with relapse-free survival** **a**–**c** *DTX1* expression is lower while *CBL* and *ITCH* expressions are normal or elevated in gastric intestinal type adenocarcinoma. Expression of *DTX1* (**a**), *CBL* (**b**), and *ITCH* (**c**) mRNA in human gastric cancer tissue versus normal^43–45^. All three databases contain patients presenting with gastric intestinal type adenocarcinoma. The numbers of patient samples in each database are indicated in Fig. 1a. *P ≤ 0.05, **P ≤ 0.01, ***P ≤ 0.001 by Student’s *t*-test (gastric intestinal type adenocarcinoma vs normal). Data were taken from Oncomine (www.oncomine.org). **d**–**f** *DTX1* expression (**d**) is mostly downregulated, whereas *CBL* (**e**) and *ITCH* (**f**) expressions are mostly increased in different human gastric cancer cell lines. The names and types of the human gastric cancer cell lines from Ooi et al.^45^ are indicated at right. **g**–**j** Higher *DTX1* expression in a gastric cancer cluster with better relapse-free survival (RFS). Kaplan–Meier plots (**g**) of two gastric cancer clusters of different relapse-free survival (RFS)^46^. *P* *=* 0.001 by the log-rank test. Analysis of *DTX1* (**h**), *CBL* (**i**), and *ITCH* (**j**) expression between C1 and C2 clusters. ***P *≤* 0.001 by Student’s *t*-test NS, not significant.

We found that expression of the ubiquitin E3 ligase *DELTEX1* (*DTX1*) is reduced in gastric adenocarcinoma tissues from patients (Fig. 1a). In contrast, expression of *ITCH* and *CBL*, the ubiquitin E3 ligases that have been shown to promote polyubiquitination and proteosomal degradation of c-FLIP^25,26^, are normal or increased in the same gastric cancer tissues (Fig. 1b, c)^43–45^. In another analysis of human gastric cancer cell lines^45^, expression of *DTX1* is reduced in most of the gastric cancer cell lines examined (Fig. 1d). Expression of *CBL* is variable in different gastric cancer cell lines, whereas the expression of *ITCH* is increased in the same group of gastric cancer cell lines (Fig. 1e, f), suggesting that the ITCH-mediated and CBL-mediated c-FLIP degradation processes are not operational in gastric cancer. Gene expression-based prognosis risk score analyses in gastric cancer have also shown that gastric cancer tissues from relapse-free survival (RFS) patients^46^ expressed higher levels of *DTX1* mRNA (Fig. 1g, h). This is in contrast to no correlation being found between the expression of *CBL* or *ITCH* and the RFS of gastric cancer patients (Fig. 1i, j). Therefore, *DTX1* is downregulated in gastric cancer tissues and *DTX1* expression is negatively correlated with gastric cancer progression.

### DTX1 promotes c-FLIP downregulation and TRAIL-induced apoptosis in gastric cancer cells

**Fig. 2:**
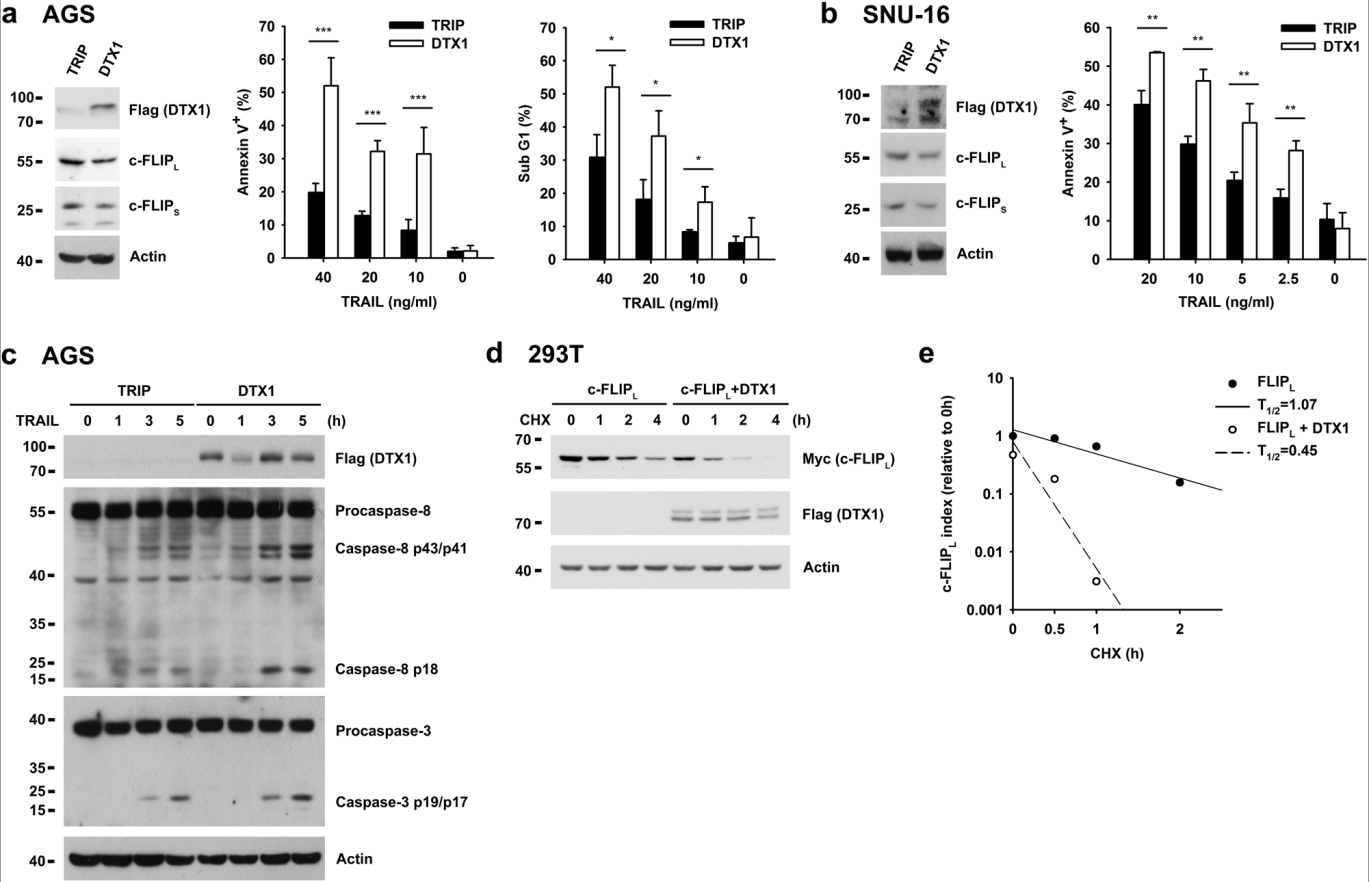
**DTX1 sensitizes TRAIL-induced apoptosis via c-FLIP downregulation** **a** DTX1 enhances TRAIL-induced apoptosis in AGS cells. The levels of Flag-DTX1, c-FLIP_L_ and c-FLIP_S_ in control (TRIP) and DTX1-expressing AGS cells were determined (left). Control and DTX1-expressing AGS cells were treated with TRAIL. Cell death, as established from the Annexin V^+^ population and sub-G1 cell fraction, were quantitated 5 and 24 h after TRAIL treatment, respectively. Values are mean ± SD of triplicates in an experiment. *P *≤* 0.05, **P *≤* 0.01, ***P *≤* 0.001. The experiment was independently repeated three times. **b** DTX1 promotes TRAIL-induced cell death in SNU-16 cells. Control (TRIP) and DTX1-expressing SNU-16 cells were treated with TRAIL at the indicated concentrations. The Annexin V^+^ population and levels of c-FLIP_L_, c-FLIP_S_, and DTX1 in control and DTX1-expressing SNU-16 cells were determined as in (**a**). Values are mean ± SD of a triplicate experiment. Experiments were independently repeated three times with similar results. **c** DTX1 enhances activation of caspase-8 and caspase-3. Control (TRIP) and DTX1-expressing AGS cells were treated with 20 ng/ml TRAIL. The levels of procaspase-8, processed caspase-8, procaspase-3, and active caspase-3 at the indicated time-points were analyzed by Western blot. The experiment was independently repeated three times with comparable results. **d**, **e** DTX1 decreases FLIP_L_ protein stability. c-FLIP_L_-Myc was transfected with or without DTX1-Flag into 293T cells. After 24 h, transfected cells were treated with cycloheximide (CHX, 50 ng/ml) for the indicated time-points. Cell lysates were prepared and the contents of c-FLIP_L_-Myc and DTX1-Flag were determined **c**. The protein levels of c-FLIP_L_ were quantitated by densitometer. The c-FLIP_L_ protein level at time zero was set as 1. The half-life of c-FLIP_L_ protein was calculated as (**d**)

DTX1 is a ubiquitin E3 ligase that is functionally analogous to ITCH and CBL in T cell anergy^40,42^. Since ITCH and CBL stimulate c-FLIP downregulation, we examined the possible effect of DTX1 on levels of c-FLIP_L_. We found that overexpression of DTX1 in human gastric adenocarcinoma AGS cells decreased the levels of c-FLIP_L_ and c-FLIP_S_ (Fig. 2a). Consistent with a reduction in c-FLIP, DTX1-expressing AGS cells were more sensitive to apoptosis induced by TNF-related apoptosis-inducing ligand (TRAIL) (Fig. 2a). In another gastric cancer cell line, SNU-16, DTX1 expression also reduced the protein levels of FLIP_L_ and c-FLIP_S_, with a concomitant increase in TRAIL-induced cell death (Fig. 2b). This effect was accompanied by enhanced activation of pro-caspase-8 into p43 and p18 in DTX1-expressing AGS cells (Fig. 2c). Enhanced formation of active caspase-3 was also found in TRAIL-stimulated DTX1-expressing AGS cells (Fig. 2c).

DTX1 expression did not affect mRNA expression of c-FLIP (Supplementary Fig. 1a), nor did it affect the levels of Mcl-1, Bcl-2, or caspase-8 (Supplementary Fig. 1b). Even though total concentrations of the TRAIL receptors DR4 and DR5 were increased in DTX1-expressing AGS cells, cell surface levels of DR4 and DR5 remained unchanged (Supplementary Fig. 1b). In contrast, DTX1 increased c-FLIP_L_ protein instability (Fig. 2d), with co-expression of DTX1 decreasing the half-life of c-FLIP_L_ protein by 50% (Fig. 2e).

SNU-16 cells are different from AGS cells due to their detectable expression levels of endogenous DTX1, allowing us to address the role of DTX1 by its knockdown. Levels of FLIP_L_ and c-FLIP_S_ proteins were increased in DTX1-knockdown SNU-16 cells, consistent with a reduction in TRAIL-induced apoptosis (Supplementary Fig. 2). Together, these results suggest that DTX1 enhances DR-induced apoptosis through downregulation of FLIP_L_.

### DTX1 interacts with c-FLIP_L_

**Fig. 3:**
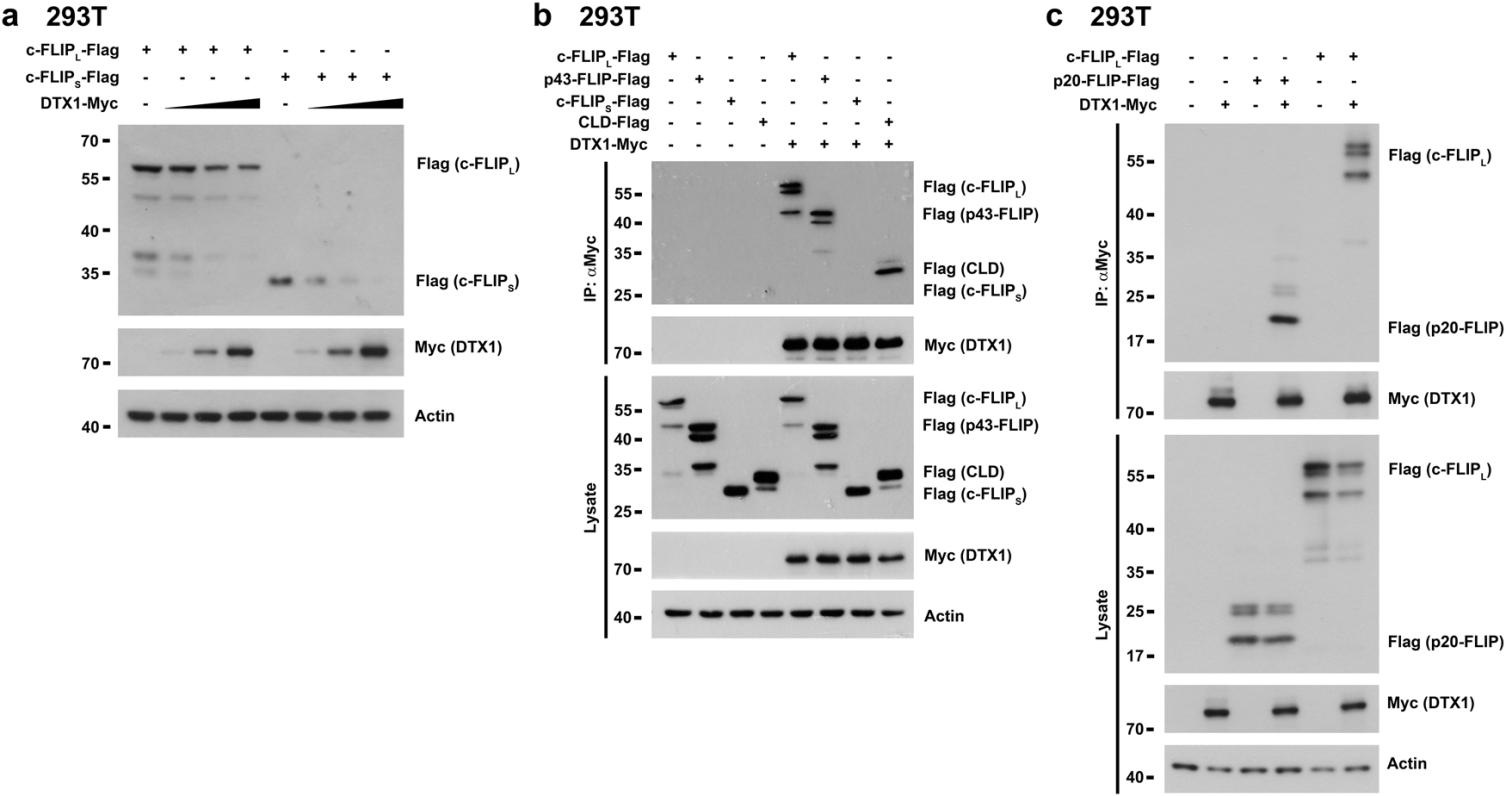
**DTX1 binds c-FLIP**_**L**_**and promotes c-FLIP**_**L**_**downregulation** **a** DTX1 promotes c-FLIP degradation. 293T cells were transfected with c-FLIP_L_-Flag (200 ng) or c-FLIP_S_-Flag (2 ng) with increasing amounts of DTX1-Myc. The levels of DTX1, c-FLIP_L_ and c-FLIP_S_ were determined 24 h after transfection. **b** The caspase-like domain of c-FLIP binds DTX1. DTX1-Myc, c-FLIP_L_, c-FLIP-p43, c-FLIP_S_ and the caspase-like domain (CLD) were transfected into 293T cells as indicated. Cell lysates were prepared 24 h later and were immunoprecipitated by anti-Myc. The presence of DTX1 and different forms of c-FLIP in the precipitates and lysates were determined by anti-Myc and anti-Flag. **c** The p20 region in the CLD of c-FLIP binds DTX1. DTX1-Myc, c-FLIP_L_, and c-FLIP-p20 were transfected into 293T cells as indicated. The levels of DTX1 and different forms of c-FLIP in lysates and anti-Myc immunoprecipitates were determined by anti-Myc and anti-Flag. Experiments (**a**–**c**) were independently repeated three times with similar results

We next examined how DTX1 regulates the stability of c-FLIP protein. We found that DTX1 promoted downregulation of c-FLIP_L_-FLAG and c-FLIP_S_-FLAG in 293T cells (Fig. 3a). Immunoprecipitation of DTX1-myc brought down c-FLIP_L_-FLAG and p43 c-FLIP_L_-FLAG, but not c-FLIP_S_-FLAG (Fig. 3b). The c-FLIP caspase-like domain (CLD) was identified as the DTX1-interacting region, evidenced by the ability of DTX1 to bind the CLD (Fig. 3b). We further demonstrated that p20 of the CLD was pulled down by DTX1 (Fig. 3c), suggesting that p20 of the CLD is the region on c-FLIP that binds DTX1. Therefore, DTX1 binds c-FLIP_L_ but does not interact with c-FLIP_S_. We then investigated how DTX1 enhances the down-regulation of c-FLIP_S_ in 293T cells (Fig. 3a). Only c-FLIP_L_ was expressed in 293T cells (Supplementary Fig. 3a). When transfected in higher amounts into 293T cells, c-FLIP_S_ was resistant to DTX1-mediated downregulation (Supplementary Fig. 3b), in agreement with the inability of c-FLIP_S_ to bind DTX1. However, c-FLIP_S_-FLAG was downregulated by DTX1 when it was co-expressed with c-FLIP_L_-FLAG (Supplementary Fig. 3c). Immunoprecipitation of DTX1-myc also pulled down c-FLIP_S_-FLAG when c-FLIP_L_-FLAG was co-expressed (Supplementary Fig. 3d). In addition, on introduction of c-FLIP_S_-FLAG alone, it was found to associate with endogenous c-FLIP_L_ in 293T cells (Supplementary Fig. 3e). These results suggest that intracellular hetero-dimerization of c-FLIP_S_ with c-FLIP_L_ leads to susceptibility of both c-FLIP_S_ and c-FLIP_L_ to DTX1-mediated downregulation in vivo (Fig. 2a, b).

### DTX1 promotes c-FLIP_L_ degradation by the endosome-lysosome-dependent pathway

**Fig. 4:**
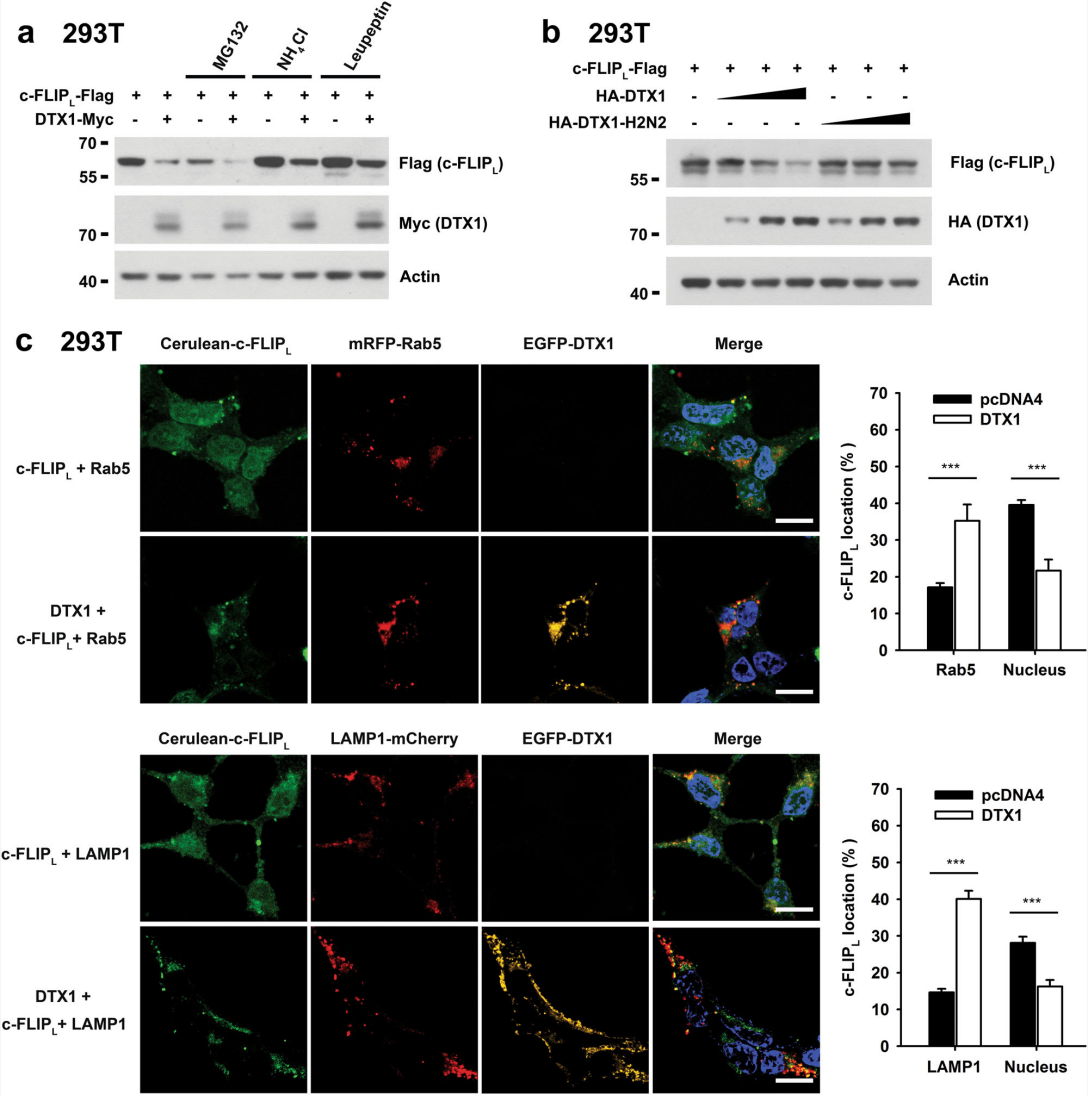
DTX1 directs c-FLIP_L_ into the endosome-lysosomal degradation pathway **a** DTX1-induced c-FLIP_L_ degradation was inhibited by NH_4_Cl and Leupeptin. DTX1-Myc and/or c-FLIP_L_-Flag were transfected into 293T cells. After 24 h, cells were untreated, or treated with MG132 (2.5 μM), NH_4_Cl (25 mM), or Leupeptin (100 μg/ml) for 8 h. The levels of c-FLIP_L_-Flag and DTX1-Myc were determined. **b** Mutation at the RING finger abolishes the ability of DTX1 to downregulate c-FLIP_L_. 293T cells were transfected with c-FLIP_L_-Flag with increasing amounts of HA-DTX1 or HA-DTX1-H2N2 (H453N, H456N). The levels of DTX1 and c-FLIP_L_ were determined 24 h after transfection. **c** DTX1 directs c-FLIP_L_ into endosome–lysosome compartments. 293T cells were transfected with mRFP-Rab5, LAMP1-mCherry, Cerulean-c-FLIP_L_, or EGFP-DTX1 as indicated. After 24 h, 293T cells were seeded onto polylysine-coated glass coverslips and allowed to attach for another 18 h. Cells were fixed and then mounted in DAPI-Fluoromount-G (Southern Biotechnology Associates). The images of EGFP, Cerulean, mRed, mCherry, and DAPI were obtained under a Zeiss LSM 780 confocal microscope (Zeiss). Experiments were independently repeated three times with similar results. Co-localization of Cerulean-c-FLIP_L_ with mRFP-Rab5 or LAMP1-mCherry in 40–50 cells was calculated using WCIF ImageJ software. Scale bars, 10 μm, ***P *≤* 0.001

DTX1-mediated c-FLIP_L_ degradation was not prevented by addition of the proteosome inhibitor MG132 (Fig. 4a), suggesting that proteasomes are not involved in this process. Addition of NH_4_Cl or leupeptin partially protected c-FLIP_L_ from DTX1-induced degradation (Fig. 4a), indicating the involvement of endosome-lysosomal pathways. To further determine the requirement of E3 ligase activity of DTX1 in c-FLIP degradation, we used DTX1 with mutation at E3 ligase active sites (H453N and H456N, H2N2) and found that DTX1-H2N2 was unable to downregulate c-FLIP_L_ (Fig. 4b).

c-FLIP_L_ alone was evenly distributed in the nucleus and cytoplasm, without apparent co-localization with the early endosome marker Rab5 (Fig. 4c). Co-expression of DTX1 and c-FLIP_L_ resulted in a significant increase in c-FLIP_L_ and Rab5 co-localization (Fig. 4c), suggesting that entry of c-FLIP_L_ into endosomal compartments is promoted by the c-FLIP_L_-DTX1 association. Similarly, c-FLIP_L_ was not located in lysosomes when expressed alone, yet the presence of DTX1 enhanced co-localization of c-FLIP_L_ with the lysosomal marker LAMP (Fig. 4c). Together, these results suggest that DTX1 directs c-FLIP_L_ into endosomal and lysosomal compartments for c-FLIP degradation.

### Geldanamycin decreases the protein level of c-FLIP and enhances TRAIL sensitivity

**Fig. 5:**
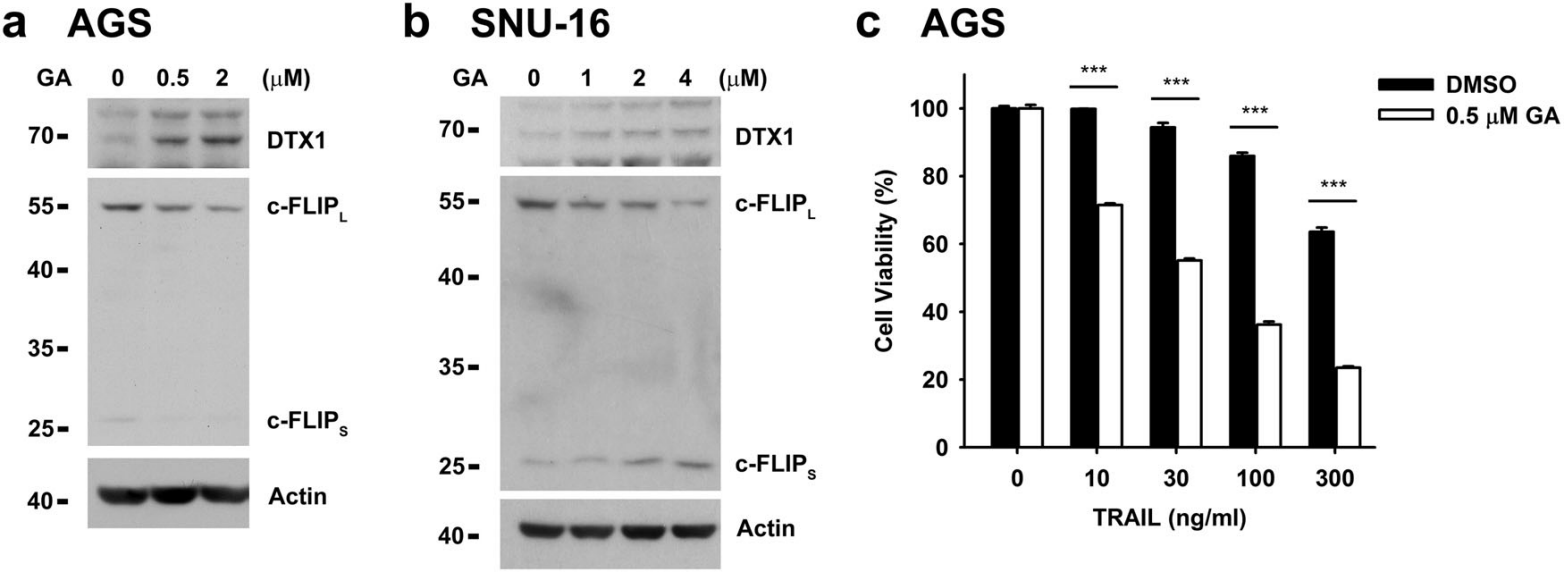
**Geldanamycin decreases the protein level of c-FLIP and enhances TRAIL sensitivity** **a**, **b** Geldanamycin (GA) promotes c-FLIP_L_ degradation. AGS cells (**a**) or SNU-16 (**b**) cells were treated with GA at the indicated concentrations for 24 h. The levels of DTX1, c-FLIP_L_, and c-FLIP_S_ were determined by immunoblotting. **c** GA sensitizes AGS cells to TRAIL-induced apoptosis. AGS cells were treated with 0.5 μM GA overnight and then incubated with TRAIL for 24 h. Cell viability was determined by MTT assay. Values are means ± standard deviations of three independent experiments. ***P *≤* 0.001

We next examined whether the DTX1-mediated downregulation of c-FLIP contributes to sensitization of cancer cells to TRAIL-induced cell death. Geldanamycin (GA) induces c-FLIP_L_ degradation and cellular apoptosis in human lung cancer cells^47^. Treatment of AGS and SNU-16 cells with GA for 24 h also resulted in c-FLIP_L_ downregulation (Fig. 5a, b). Addition of GA also enhanced the ability of TRAIL to induce cell death in AGS cells (Fig. 5c). We found that GA treatment led to increased DTX1 protein levels in AGS and SNU-16 cells (Fig. 5a, b). Notably, GA treatment did not affect the expression of DTX1 or c-FLIP mRNA (Supplementary Fig. 4).

**Fig. 6:**
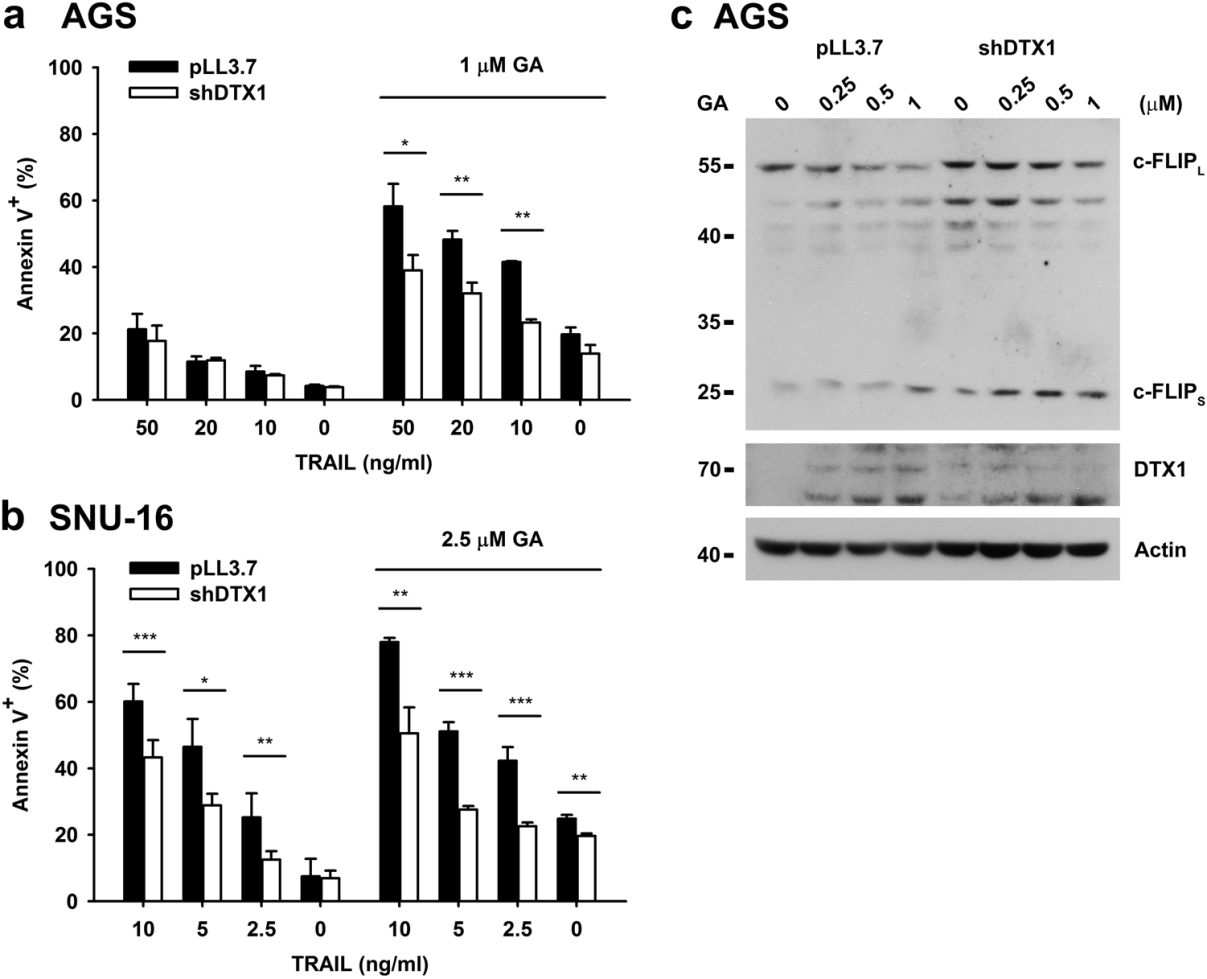
**Geldanamycin enhances TRAIL-induced apoptosis through DTX1-mediated c-FLIP**_**L**_**degradation** **a**, **b** DTX1 knockdown reduces GA-enhanced TRAIL sensitivity. Control (pLL3.7) and DTX1-knockdown (shDTX1) AGS cells (**a**) or SNU-16 cells (**b**) were incubated with GA overnight followed by TRAIL treatment for 5 h. Apoptosis was determined by Annexin V/PI staining. Values are means ± standard deviations of an experiment with triplicate (**a**) or three independent experiments (**b**). *P *≤* 0.05, **P *≤* 0.01, ***P *≤* 0.001. Experiment (**a**) was repeated three times with similar results. **c** DTX1-knockdown attenuates GA-induced c-FLIP_L_ degradation. Control (pLL3.7) and DTX1-knockdown (shDTX1) AGS cells were treated with GA overnight. The levels of DTX1, c-FLIP_L_, and c-FLIP_S_ were determined. Experiments (**c**) was independently repeated three times with similar results

We then examined whether DTX1 was involved in GA-induced TRAIL sensitivity and c-FLIP degradation. DTX1-knockdown did not affect apoptosis induced by TRAIL alone in AGS cells, likely due to low DTX1 expression (Fig. 6a), but DTX1-deficiency reduced apoptosis triggered by TRAIL plus GA (Fig. 6a). The celldeath triggered by the combination of TRAIL and GA was similarly attenuated by DTX1-knockodwn in SNU-16 cells (Fig. 6b). GAinduced c-FLIP degradation was prevented by DTX1-knockdown in AGS cells (Fig. 6c). These results suggest that the GA-induced c-FLIP degradation was partly attributable to the induction of DTX1, leading to enhanced sensitivity of gastric cancer cells to TRAIL treatment. Together, our results suggest that increased DTX1 enhances TRAIL-triggered apoptosis in gastric cancer cells partly by downregulation of c-FLIP.

### DTX1 enhances FasL-induced and TRAIL-induced apoptosis in T cells

**Fig. 7:**
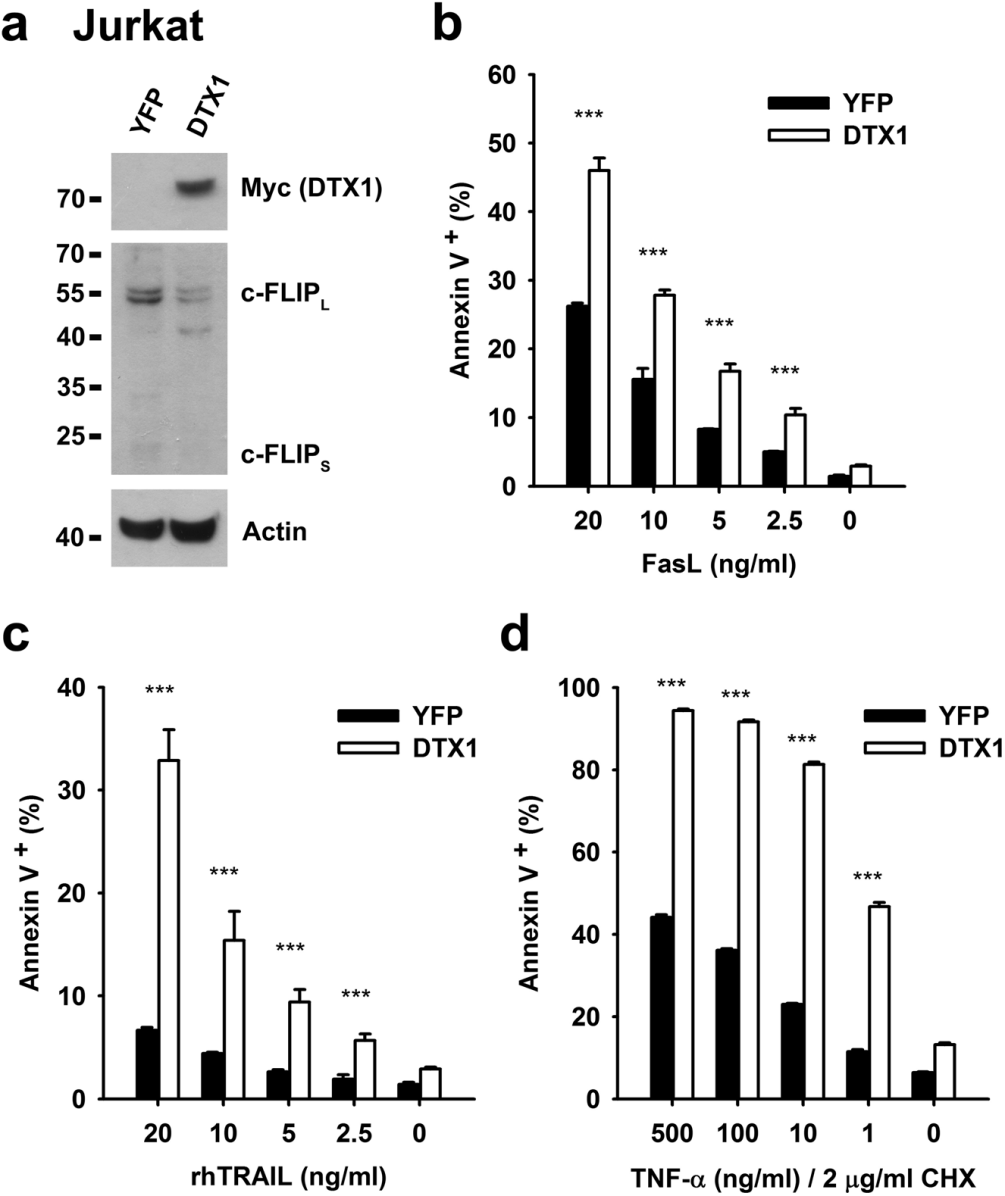
**DTX1-overexpression enhances FasL-induced, TRAIL-induced and TNF-α-induced apoptosis in Jurkat cells** **a** Reduced c-FLIP levels in DTX1-overexpressing Jurkat cells. The levels of c-FLIP_L_, c-FLIP_S_, and DTX1(Myc) in control (YFP) and DTX1-expressing Jurkat cells were determined. **b**–**d** Control (YFP) and DTX1-expressing Jurkat cells were treated with FasL **b**, TRAIL **c**, or TNF-α **d** at the indicated concentrations. The extent of apoptosis was analyzed 6 h later by flow cytometry with Annexin V staining. Data are mean ± SD of a triplicate experiment. Experiments were independently repeated three times with similar results. ***P *≤* 0.001. The experiment was independently repeated twice with comparable results

The involvement of DTX1 in c-FLIP degradation is not limited to gastric cancer cells. We used *Dtx1*^*−/−*^ T cells to examine the role of DTX1 in c-FLIP downregulation. c-FLIP was barely detectable in naïve WT T cells or *Dtx1*^*−/−*^ T cells. TCR stimulation induced the expression of c-FLIP, and DTX1-deficiency increased CD3-triggered c-FLIP expression (Supplementary Fig. 5). Activated *Dtx1*^*−/−*^ T cells were more resistant to FasL-induced apoptosis. We further explored whether overexpression of DTX1 increased the sensitivity of T lymphoma to death receptor-induced cell death by targeting c-FLIP_L_ for degradation. DTX1 expression reduced the levels of c-FLIP in Jurkat cells (Fig. 7a). Increased DTX1 expression was indeed accompanied by enhanced FasL-induced apoptosis and TRAIL-triggered cell death in Jurkat cells (Fig. 7b, c). In addition, increased DTX1 expression promoted TNF-α-induced apoptosis in Jurkat cells in the presence of cycloheximide (Fig. 7d). Thus, DTX1 promotes DR-induced cell death in normal T cells and in T lymphoma. DTX1-promoted c-FLIP instability represents one of the physiological mechanisms that regulate c-FLIP protein.

## Discussion

Here, we report a new finding that DTX1 is an E3 ligase for c-FLIP in gastric cancer cells. The presence of DTX1 profoundly reduced the protein stability of c-FLIP (Fig. 2d, e). We have previously demonstrated that DTX1, similar to Cbl-b and Itch, promotes PKCθ and PLC-γ degradation in anergic Th1 cells^42^. In addition, as for Cbl-b and Itch^39^, DTX1 induced PKCθ degradation by directing it to the endocytic pathway for lysosomal degradation. We illustrate that DTX1 promotes c-FLIP_L_ protein downregulation by similarly directing c-FLIP_L_ into the endosome-lysosomal pathway for degradation (Fig. 4). Consequently, DTX1 expression enhanced the susceptibility of gastric cancer cells to TRAIL-induced apoptosis (Fig. 2a, b), whereas DTX1 downregulation increased the resistance to TRAIL-triggered cell death (Supplementary Fig. 2).

We have demonstrated that DTX1 interacts with c-FLIP_L_ but not with c-FLIP_S_, and reveal that the p20 region of the caspase-like domain in c-FLIP_L_ is the DTX-association domain (Fig. 3b, c). Binding of DTX1 to c-FLIP_L_ leads to c-FLIP_L_ degradation. Notably, expression of DTX1 led to a reduction of both c-FLIP_L_ and c-FLIP_S_ levels in AGS, SNU-16, and Jurkat cells (Figs 2a, b and 7a). A likely cause for the downregulation of c-FLIP_S_ in these cancer cells is that c-FLIP_L_ is hetero-dimerized with c-FLIP_S_ in vivo and that the binding of DTX1 to c-FLIP_L_ induces the degradation of c-FLIP_L_-c-FLIP_S_ dimers. c-FLIP_S_ was associated with the endogenous c-FLIP_L_ in 293T cells (Supplementary Fig. 3e). This is further supported by the fact that DTX1 is co-expressed with c-FLIP_L_ and c-FLIP_S_ dimers in 293T cells, resulting in downregulation of both c-FLIP_L_ and c-FLIP_S_ (Fig. 3a, Supplementary Fig. 3c), and by the observation that c-FLIP_S_ is associated with DTX1 under co-expression of c-FLIP_L_ (Supplementary Fig. 3d).

DTX1 was downregulated in most of our gastric cancer samples (Fig. 1). Increased expression of c-FLIP in DTX1-deficient cells suggests that DTX1 downregulation confers resistance to TRAIL-induced apoptosis in gastric cancer cells. This finding, together with the negative correlation between cancer prognoses and DTX1 expression levels (Fig. 1g, h), suggest that manipulation of DTX1 could be beneficial in therapies against gastric cancer. We propose that reagents that can stimulate the expression of DTX1 may enhance the susceptibility of gastric cancer to TRAIL treatment.

Interaction with HSP90 contributes to the protein stability of c-FLIP. HSP90 also recruits c-FLIP to DISC to inhibit TRAIL-induced cell death^48^. HSP-90 inhibitors are known to exhibit inhibitory effects on multiple signaling pathways in cancer cells^49^. These inhibitors act synergistically with TRAIL to induce cell death in various cancers^50–52^. As reported for gastric cancer^53^, we found that addition of GA augmented TRAIL-induced apoptosis (Fig. 5). We have further demonstrated that DTX1 partly mediates the enhancing effect of GA on TRAIL-induced apoptosis (Figs 5 and 6). Knockdown of DTX1 partly reversed the enhancing effect of GA in AGS and SNU-16 cells (Fig. 6). Even though the exact molecular mechanism requires further investigation, it should be noted that other deltex family members (DTX2, DTX3, DTX4) are also associated with HSP90.

Our results suggest variability in the recruitment of a specific E3 ligase and in the mechanistic process of c-FLIP degradation in different cell types. Itch mediates c-FLIP_L_ ubiquitination and degradation in conjunction with JNK signaling in hepatocytes and fibroblasts^25^. CBL has been shown to promote c-FLIP_S_ degradation in non-small cell lung carcinoma cells via mTORC2-dependent signaling^26^. Here, we found that DTX1 enhances c-FLIP_L_ downregulation in gastric cancer cells and lymphomas via an endosome-lysosomal pathway. Given the vast heterogeneity between different tumors, selection of E3 ligases for c-FLIP degradation is likely determined by the availability of the given E3 ligase and the signaling within tumor microenvironments. We also illustrate that c-FLIP protein levels are regulated by DTX1 in normal T cells (Supplementary Fig. 5), suggesting that DTX1 represents one of the physiological mechanisms controlling c-FLIP stability. It should be noted that the effect of DTX1-deficiency was not overwhelming (Supplementary Figs. 2 and 5), suggesting that DTX1 contributes to but is unlikely to be the only regulator of c-FLIP protein instability.

In summary, we identify DTX1, but not CBL or ITCH, as the E3 ligase regulating c-FLIP protein stability in gastric cancer cells. In addition, we show that DTX1 joins ITCH and CBL as another E3 ligase controlling c-FLIP under physiological conditions. Different c-FLIP degradation pathways are likely to have cancer type-dependent mechanisms, yet to be determined. We have also demonstrated that gastric cancer cells, which are relatively resistant to TRAIL treatment, become susceptible to TRAIL-induced cell death in the presence of DTX1. Our results suggest that a combination of TRAIL with compounds that increase DTX1 expression could be a new approach for gastric cancer therapy.

## Materials and methods

### Reagents

Recombinant human TRAIL and anti-His were obtained from R&D Systems (Minneapolis, MN). MG132, propidium iodide (PI), 3-(4,5-dimethyl-thiazol-2-yl)-2,5-diphenyl tetrazolium bromide (MTT), and anti-Flag-HRP were purchased from Sigma (St. Louis, MO). Rabbit anti-DTX1 polyclonal antiserum against GST-Deltex1 was generated as described^9^. The following antibodies were obtained from Santa Cruz Biotech (Santa Cruz, CA): anti-caspase-3 (H-277), anti-DR4 (H130), anti-DR5 (N-19), anti-Mcl-1 (S-19), and anti-Bcl-2 (N-19). Annexin V-Cy5 was obtained from Biovision (Mountain View, CA). Anti-Myc (9B11), anti-caspase-8 (1C12), and anti-active caspase-3 (D175) were purchased from Cell Signaling (Beverly, MA). Anti-actin (clone C4) and anti-β-tubulin (clone AA2) were purchased from Millipore (Temecula, CA). Anti-human FLIP mAb (NF6) was purchased from AdipoGen (San Diego, CA). Horseradish peroxidase-conjugated secondary antibodies were purchased from Jackson ImmunoResearch Laboratories. WesternBright ECL HRP substrate was obtained from Advansta Corporation (Menlo Park, CA). Dapi-Fluoromount-G^TM^ was obtained from SouthernBiotech (Birmingham, AL). Protein G Mag Sepharose^TM^ Xtra was obtained from GE Healthcare (Piscataway, NJ).

### Analysis of cancer gene microarray database

The publicly-accessible Oncomine cancer microarray database (Compendia Biosciences; Ann Arbor, MI, USA; www.oncomine.com) was used to examine the expression of *DTX1* in human gastric cancer tissue and cancer cell lines. D’Errico et al. (GEO accession GSE13911)^43^, Förster et al. (GEO accession GSE22377)^44^, and Ooi et al. (GEO accession GSE15459)^45^ datasets were used to compare *DTX1*, *ITCH,* and *CBL* expression levels between cancer and normal tissues. The Ooi et al. (GEO accession GSE15455)^45^ dataset was used to compare expression levels of *DTX1*, *ITCH*, and *CBL* among a panel of cell lines that represent adenocarcinoma, adenosquamous carcinoma, choriocarcinoma, and tubular adenocarcinoma of gastric cancer.

Hierarchical clustering analysis of gene expression data from 65 human primary tumor tissue samples and six gastrointestinal stromal tumor tissue samples was used to determine gene expression with gastric cancer outcomes^46^. Primary microarray data (GEO accession GSE13891) were used to compare *DTX1*, *ITCH*, and *CBL* expression levels between C1 and C2 clusters.

### Cell culture and transfection

AGS and SNU-16 cells were cultured in RPMI 1640 medium with 10% FCS (Life Technologies-Invitrogen), 10 mM glutamine, 100 U/ml penicillin, 100 μg/ml streptomycin, and 20 mM 2-ME. DMEM (Life Technologies) was used in the culture of 293T cells. Transfection of 293T cells was performed by using OmicsFect In Vitro Transfection Reagent (Omics Biotechnology, Taiwan).

### DTX1 overexpression and knockdown

For overexpression of DTX1, mouse DTX1 cDNA (a gift from Dr. Hideyuki Okano, Keiko University, Tokyo, Japan) was tagged with FLAG and subcloned into pTRIP-IRES-GFP to generate pTRIP-DTX1-IRES-GFP. 293T cells were transfected with pTRIP-IRES-GFP or pTRIP-c-FLIP-IRES-GFP, psPAX2, and pMD2G, and the lentivirus-containing culture supernatants were harvested 48 h after transfection. AGS and SNU-16 cells were infected with recombinant lentivirus, and GFP-expressing cells were isolated by sorting on a FACSAriaII SORP system (BD Biosciences). The c-FLIP p20 (198–376) fragment was isolated by PCR using forward primer 5′ ATG TCA AAT AAC TTC AGG CTC C and reverse primer 5′ ATC CAC CTC CAA GAG GCT GC. A full-length 2XHA-DTX1 (H453N and H456N, H2N2) mutant was generated^40^ and subcloned into pcDNA4 for expression in 293T cells.

For DTX1 knockdown, human DTX1-specific shRNA was subcloned into pLentiLox vector (pLL3.7; a gift from Dr. Luk Van Parijs, Massachusetts Institute of Technology, Cambridge, MA). The sequence of the human DTX1-specific shRNA was 5′-GAA GTT CAC CGC AAG AGG A-3′. Lentiviruses were harvested from culture supernatants of 293T cells transfected with pLL3.7 or pLL3.7-shDTX1, psPAX2, and pMD2.G. AGS and SNU-16 cells were infected with recombinant lentivirus, and GFP-expressing cells were sorted 48 h post-infection on a FACSAriaII SORP system (BD Biosciences, Mountain View, c-FLIP mouse [18] was a gift of Dr. You-Wen He (Duke University Medical Center, Durham NC). Mice with T cell-specific knockout of c-FLIP were generated by crossing of c-FLIP mouse with Cd4-Cre mouse. Mouse experiments were conducted with approval from the Institutional Animal Care and Use Committee, Academia Sinica. CA).

### Apoptosis and cell viability assays

Several different methods were used to measure apoptosis and viability. After TRAIL treatment for 5–6 h, cells were stained with Annexin V-Cy5 and propidium iodide (PI), and annexin V^+^ populations were quantified by flow cytometry. After TRAIL treatment for 24 h, cells were stained with PI in hypotonic solution (50 mg/ml PI, 0.1% sodium citrate, 0.1% Triton X-100) overnight at 4 °C. Fractions of cells with sub-G1 DNA content were quantified using CellQUEST software on a FACSCalibur flow cytometer (BD Biosciences). For viability determination, AGS cells were plated in 96-well plates for 24 h before treatment. After TRAIL treatment, the cells were incubated with 0.5 mg/ml MTT in complete medium for 2 h. The surviving cells converted MTT to generate a purple-colored formazan product when dissolved in dimethyl sulfoxide (DMSO). The intensity of formazan product was measured by absorbance at 490 nm using SOFTmax PRO 4.3.1 LS software accompanying an Emax microtiter plate reader (Molecular Device, Sunnyvale, CA). Cell viability was calculated by dividing the absorbance of treated cells by that of the control. For measurement of caspase activation, control and DTX1-expressing cells were treated with 20 ng/ml TRAIL. The levels of procaspase-8, procaspase-3, processed caspase-8 (p43/41, p18) and processed caspase-3 (p19/17) were determined by immunoblots.

### Surface staining

For staining of cell surface DR4 and DR5, AGS cells were incubated with anti-DR4 or anti-DR5 in PBS containing 2% FBS for 2 h. After washing with PBS, cells were stained with allophycocyanin-conjugated anti-Rabbit IgG or anti-Goat IgG, and analyzed on a FACSCalibur flow cytometry system.

### Quantitative PCR

Total RNA from AGS cells was isolated using TRIzol (Invitrogen). cDNAs were prepared and analyzed for the expression of DTX1, c-FLIP_L_ and c-FLIP_S_ on a LightCycler 480 Real-Time PCR System (Roche). The PCR protocol was 95 °C for 10 min, followed by 45 cycles of 95 °C for 10 s, 60 °C annealing for 10 s, and 72 °C extension for 8 s. The PCR primers were: human DTX1, forward, 5′CAG CCG CCT GGG AAG ATG GAG TT-3′ and reverse, 5′-TGG ATG CCT GTG GGG ATG TCA TAG AC-3′; human c-FLIP_L_, forward, 5′-CCT AGG AAT CTG CCT GAT AAT CGA-3′ and reverse, 5′-TGG GAT ATA CCA TGC ATA CTG AGA TG-3′; human c-FLIP_S_, forward, 5′-GCA GCA ATC CAA AAG AGT CTC A -3′ and reverse, 5′-ATT TCC AAG AAT TTT CAG ATC AGG A-3′.

### Immunoprecipitation

Cells were washed and lysed in whole cell extract (WCE) buffer (25 mM HEPES pH 7.4, 300 mM NaCl, 1.5 mM MgCl_2_, 0.2 mM EDTA pH 8.0, 0.1% Triton X-100, 0.5 mM DTT, 1× Complete protease inhibitor (EDTA-free, Roche)). Two hundred and fifty microgram whole cell lysate was incubated with antibodies, as indicated in each figure, on a rotating shaker overnight at 4 °C. Five microliter of Protein G Mag Sepharose was added and the samples were incubated at 4 °C for 1 h. The Protein G Mag Sepharose was washed repeatedly with 1 ml of WCE buffer. The beads were mixed with 1 × SDS-PAGE sample buffer and boiled at 100 °C for 5 min. Pull-down complex and total lysates were analyzed in 10% SDS-PAGE and transferred to polyvinylidene difluoride (PVDF) membranes (Millipore). The membranes were blocked with 5% skimmed milk for 1 h and probed with an appropriate antibody, followed by incubation with a HRP-conjugated secondary antibody. The protein bands were visualized by the ECL detection system.

### Protein stability determination

c-FLIP_L_-Myc was transfected with or without DTX1-Flag in 293T cells. Cells were treated with cycloheximide (CHX, 50 ng/ml) for the indicated timeframes. Cell lysates were prepared and contents of c-FLIP_L_-Myc and DTX1-Flag were quantitated. The direct effect of DTX1 on c-FLIP_L_ protein levels was examined by transfecting 293T cells with c-FLIP_L_ or c-FLIP_S_ in the presence of increasing amounts of DTX1. The levels of DTX1, c-FLIP_L_ and c-FLIP_S_ were determined 24 h after transfection. To identify the inhibitor of c-FLIP degradation, DTX1-Myc and/or c-FLIP_L_-Flag were transfected into 293T cells, and cells were treated with or without MG132 (2.5 μM), NH_4_Cl (25 mM), or leupeptin (100 μg/ml). The levels of c-FLIP_L_-Flag and DTX1-Myc were determined 8 h later.

### Fluorescence protein constructs and confocal imaging

pmRFP-Rab5 was obtained from Addgene (Addgene plasmid 14437). pcDNA4-EGFP-DTX1 and pcDNA4-LAMP1-mCherry were generated as described previously^9^. To generate pcDNA4-cerulean-c-FLIP_L_, cerulean fragments were amplified by PCR from Cerulean-GalT (Addgene plasmid 11930) with HindIII/EcoRI and cloned into pcDNA4-c-FLIP_L_-Myc with HindIII/EcoRI to create a translational fusion construct.

293T cells (1 × 10^6^) transduced with mRFP-Rab5 or LAMP1-mCherry were transfected with cerulean-c-FLIP_L_ and EGFP-DTX1. 24 h after transfection, cells were re-seeded on a 22 × 22 mm glass coverslip and allowed to attach for another 18 h. Cells were fixed in 2% paraformaldehyde in PBS for 15 min at 37 °C and permeabilized with 0.3% Triton X-100 for 10 min at room temperature. Cells were mounted in Dapi-Fluoromount-G^TM^ (SouthernBiotech, Birmingham, AL) and observed under a Zeiss LSM 780 confocal microscope.

### Image acquisition

Images were observed under a Zeiss LSM 780 confocal microscope with a plan-Apochromat 63×/1.4 Oil DIC objective lens at room temperature. Samples were mounted in Dapi-Fluoromount-G^TM^. EGFP and Cerulean fluorescence proteins were excited by an argon laser (488 nm). EGFP fluorescence was collected in the range of 490 to 550 nm. Cerulean fluorescence was collected in the range of 445 to 489 nm. mRFP and mCherry fluorescence proteins were excited by a HeNe laser (594 nm) and emissions were collected with a 545 nm long-pass filter. DAPI-bound DNA was excited by a diode laser (405 nm) and fluorescence was collected in the range of 405 to 470 nm. The pinholes were as follows: Ch1–55 μm (blue), Chs1–54 μm (orange), Ch2–55 μm (red), and Chs1–55 μm (green). Images were acquired by a digital AxioCam (Zeiss) microscope camera using Carl Zeiss software Zen 2.1 (black).

## Electronic supplementary material

Supplementary Figures
