# Supplementary material for: c-FLIP is a target of the E3 ligase deltex1 in gastric cancer
Source: Cell Death Dis. 2018 Jan 26;9(2):135. doi: 10.1038/s41419-017-0165-6 (PMC5833402; doi:10.1038/s41419-017-0165-6)
Supplement: Supplementary file 1 — Supplementary Figures [file 41419_2017_165_MOESM1_ESM.docx]

**
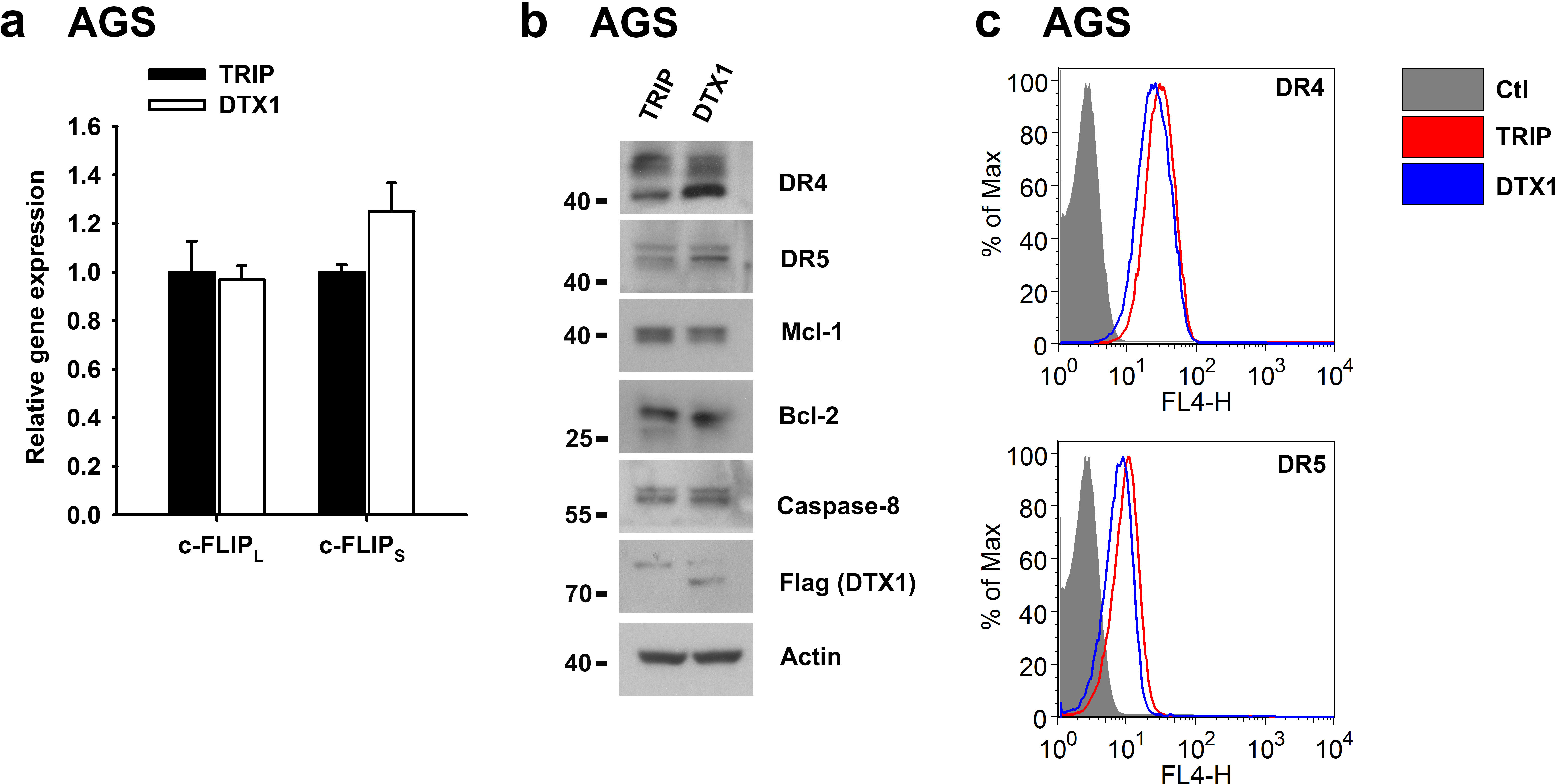
**

**Supplementary Figure 1. *DTX1* overexpression does not affect the expression of many apoptosis-associated molecules in AGS cells.** (**a**) DTX1 does not affect the expression of c-FLIP_L_ and c-FLIP_S_ mRNA in AGS cells. RNA from TRIP (control) and DTX1-expressing AGS cells was isolated, and c-FLIP_L_ and c-FLIP_S_ mRNA expression was determined by qPCR. mRNA levels were normalized against GAPDH and are expressed as mean ± SE (n=3). (**b**) Expression of DR4, DR5, caspase-8, Bcl-2, and Mcl-1 in DTX1-expressing AGS cells. Total cell lysates from control and DTX1-expressing AGS cells were analyzed for the expression of DR4, DR5, caspase-8, Bcl-2, and Mcl-1 proteins. (**c**) DTX1 does not affect DR4 and DR5 expression on AGS cell surfaces. The levels of cell surface DR4 and DR5 on TRIP- and DTX1-expressing AGS cells were determined by flow cytometry. Red line, mock (TRIP); blue line, DTX1-transduced (DTX1); shadowed curve, secondary Ab-only control (Ctl).


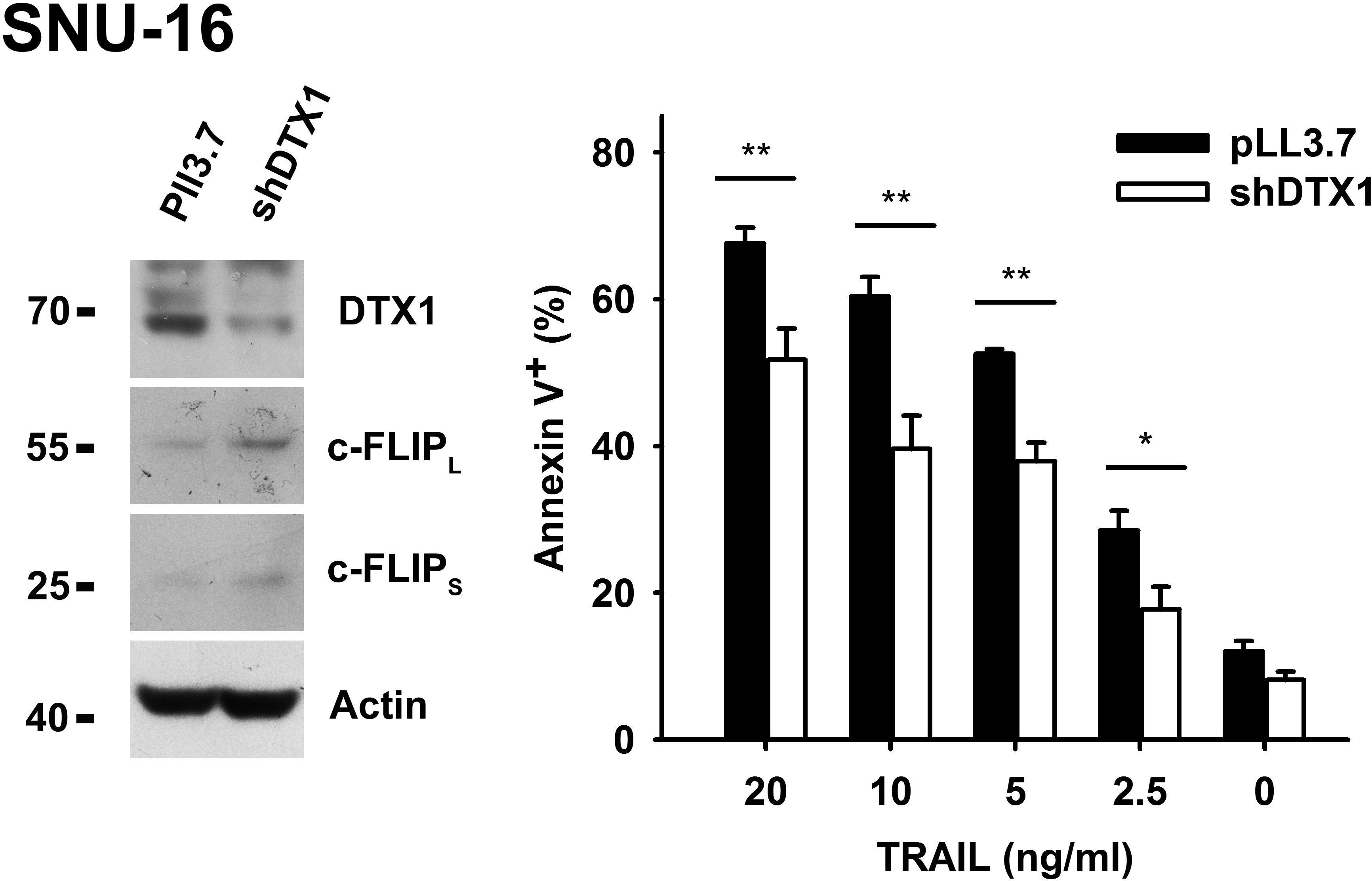


**Supplementary Figure 2. DTX1-knockdown attenuates TRAIL-induced apoptosis in SNU-16 cells.** Control (pLL3.7) and DTX1-knockdown (shDTX1) SNU-16 cells were treated with TRAIL for 5 h. Cell death was determined by Annexin V/PI staining. Expression of c-FLIP_L_ and c-FLIP_S_ was assessed by immunoblotting. The means and standard deviations were calculated from three independent experiments, each conducted in triplicate. **P ≤* 0.05, ***P ≤* 0.01.

**
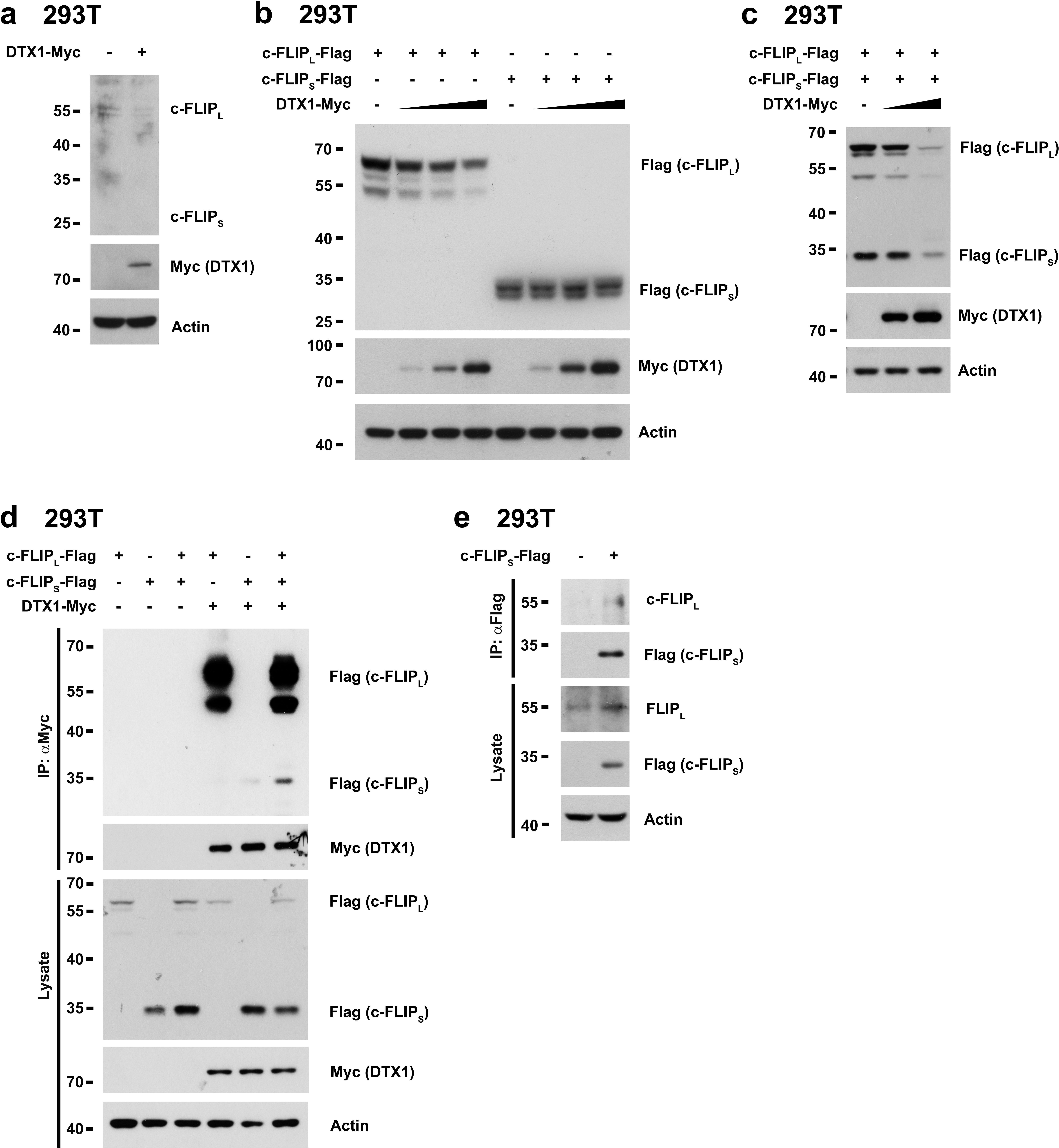
**

**Supplementary Figure 3. Interaction with** **c-FLIP_L_ renders c-FLIP_S_ susceptible to DTX1-mediated downregulation. (a)** Expression of c-FLIP_L_ but not c-FLIP_S_ in 293T cells. 293T cells were transfected with or without DTX1-Myc, and c-FLIP_L_ and c-FLIP_S_ levels were determined. **(b)** Excess c-FLIP_S_ expression is resistant to DTX1-induced downregulation. 293T cells were transfected with c-FLIP_L_-Flag, c-FLIP_S_-Flag and DTX1 as in Figure 3a, except that 1 μg of c-FLIP_L_-Flag or 100 ng of c-FLIP_S_-Flag was used. The levels of DTX1, c-FLIP_L_ and c-FLIP_S_ were determined 24 h after transfection. (**c**) DTX1 promotes c-FLIP_S_ degradation in the presence of c-FLIP_L_. 293T cells were transfected with c-FLIP_L_-Flag and c-FLIP_S_-Flag with increasing amounts of DTX1-Myc. The levels of DTX1, c-FLIP_L_ and c-FLIP_S_ were determined 24 h after transfection. (**d**) Association of DTX1 with c-FLIP_S_ in the presence of c-FLIP_L_. DTX1-Myc, c-FLIP_L_, and c-FLIP_S_ were transfected into 293T cells as indicated. Cell lysates were prepared 24 h later and were immunoprecipitated by anti-Myc. The presence of c-FLIP_L_ and c-FLIP_S_ in the precipitates and lysates were determined. (e) Transfected c-FLIP_S_-Flag binds endogenous c-FLIP_L_. 293T cells were transfected with c-FLIP_S_-Flag, and cell lysates were immunoprecipitated by anti-Flag. The presence of endogenous c-FLIP_L_ in the precipitates and lysates were determined by anti-c-FLIP (NF6). Experiments (a-e) were independently repeated three times with similar results.


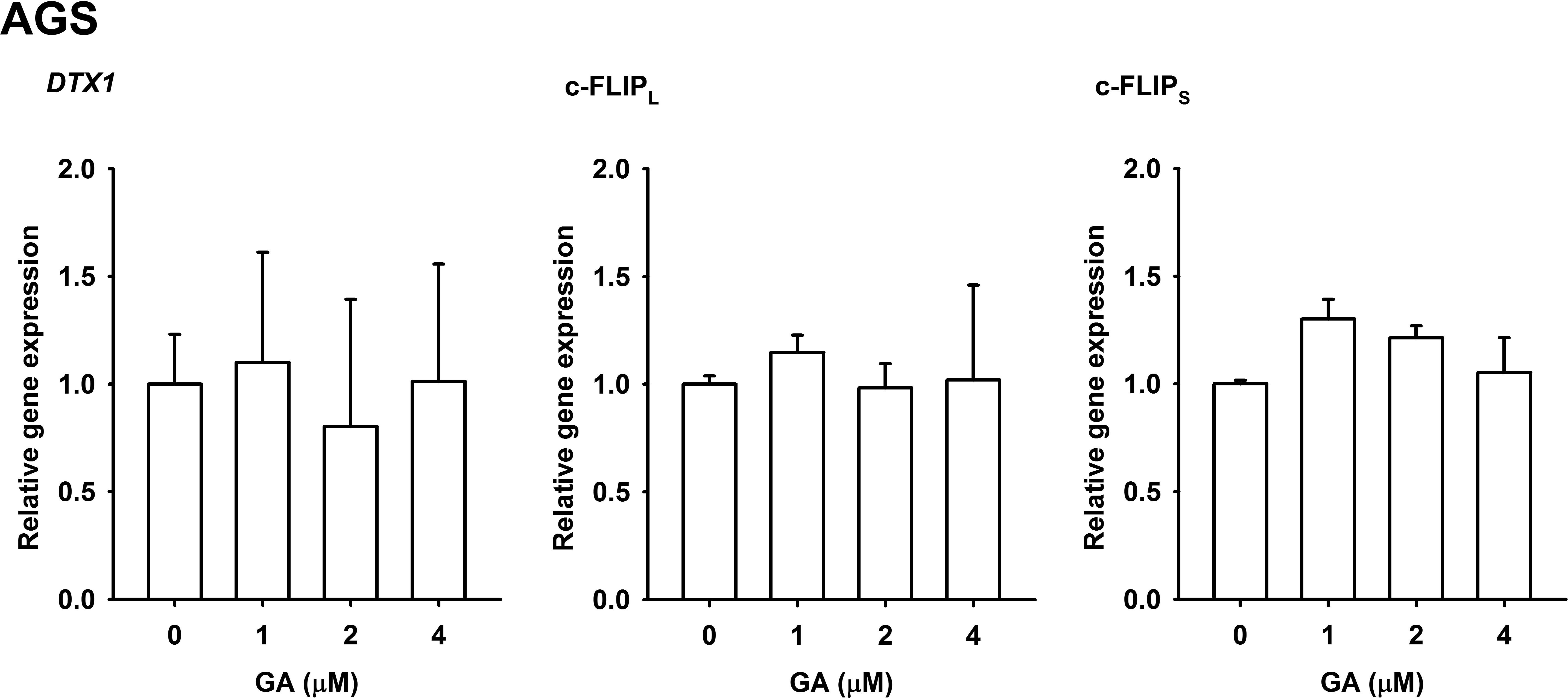


**Supplementary Figure 4. GA does not affect the expression of *DTX1* or c-FLIP mRNA.** AGS cells were treated with 2 μM GA overnight. RNA was harvested from TRIP- and DTX1-expressing AGS cells. *DTX1*, c-FLIP_L_ and c-FLIP_S_ mRNA expression was determined by quantitative PCR.

**
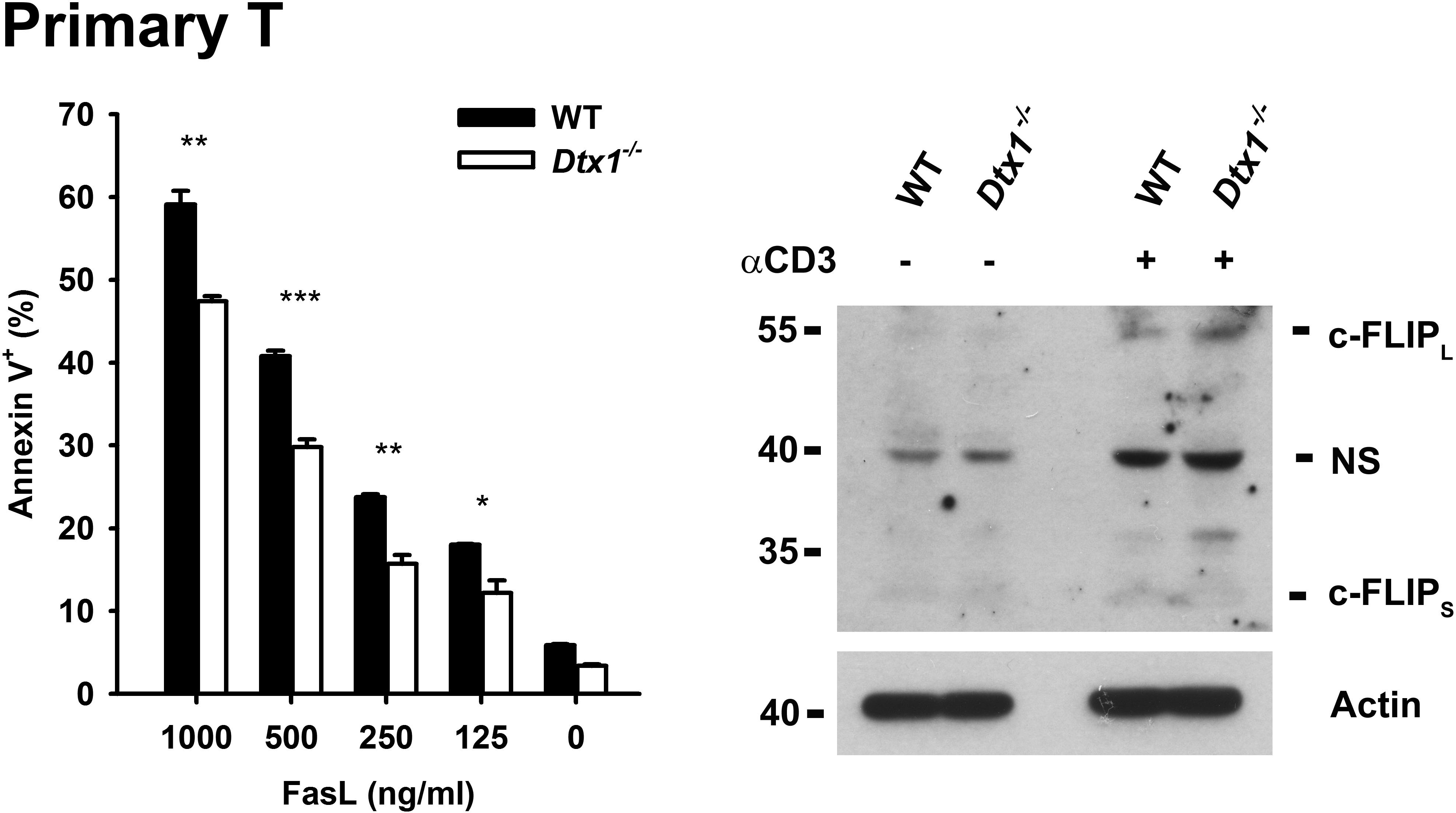
**

**Supplementary Figure 5. DTX1-deficiency reduces FasL-induced apoptosis in normal T cells.**  T cells were purified from WT and *Dtx1^-/-^* mice, and were activated with anti-CD3/CD28 for 24 h, incubated in IL-2 for 2 days, followed by stimulation with FasL at the indicated concentrations. Cell death was quantitated (left), and expression of c-FLIP before and after CD3 stimulation was determined (right). Values (left) are mean ± SD of a triplicate experiment. Experiments were independently repeated three times with similar results. **P ≤* 0.05, ***P ≤* 0.01, ****P ≤* 0.001.
